# FPGA-based acceleration of MRI registration: an enabling technique for improving MRI-guided cardiac therapy

**DOI:** 10.1186/1532-429X-16-S1-W11

**Published:** 2014-01-16

**Authors:** Ka-Wai Kwok, Gary CT Chow, Thomas CP Chau, Yue Chen, Shelley H Zhang, Wayne Luk, Ehud J Schmidt, Zion T Tse

**Affiliations:** 1 College of Engineering, University of Georgia, Athens, Georgia, USA; 2 Computing, Imperial College London, London, UK; 3 Radiology, Brigham and Women's Hospital, Harvard, Boston, Massachusetts, USA

## Background

Quantification of edema and scar maps with cardiac MR images (cMRIs) enables effective Radiofrequency Ablation (RFA) of arrhythmias during the Electrophysiology (EP) procedure [1]. This demonstrates the paramount advantage over the EP catheterization under X-ray and ultrasound guidance. High-contrast and resolution cMRIs can be obtained preoperatively as a EP roadmap for surgical planning of RFA, whilst real-time MRI (rt-MRI) can be used to guide catheterization and update the cMRI model [2] to provide intraoperative visualization of a 3D vascular map. A fast and efficient technique of non-rigid image co-registration is required. Although feature-based registration methods can be rapidly processed by computing sparse features, the outcome is sensitive to blurred images with artifacts that happens regularly in low-resolution rt-MRI, causing significant errors in feature detections. With the use of Field-programmable Gate Array (FPGA), we hypothesized that novel data structure and architecture of memory access can allow robust registration based on comparison of image intensity patterns, thus fulfilling the real-time requirements for clinical practice.

## Methods

Acquiring image gradient is a common step in intensity-based registration methods [3] (e.g. Demons [4]), but also the primary computation bottleneck. Image gradient computation requires information of pixel/voxel neighborhood, leading to large amount of non-coalesced memory accesses and floating point operations. A customized FPGA-based computation kernel of Demons is proposed. Multiple pixel/voxel processing units (PUs) are placed in the FPGA. Each has its own pixel/voxel memory. Input pixels/voxels are processed as a data stream that propagate via the kernel. The workloads are then distributed to the PUs such that neighboring gradients are connected by neighboring PUs, hence memory bandwidth is further reduced. Rapid computation of image registration is achieved by 1) the highly-customized PUs; 2) the parallelism of multiple PUs and pixel/voxel memories; and 3) bandwidth reduction through inter-PUs information exchange channels.

## Results

**Figure 1:**
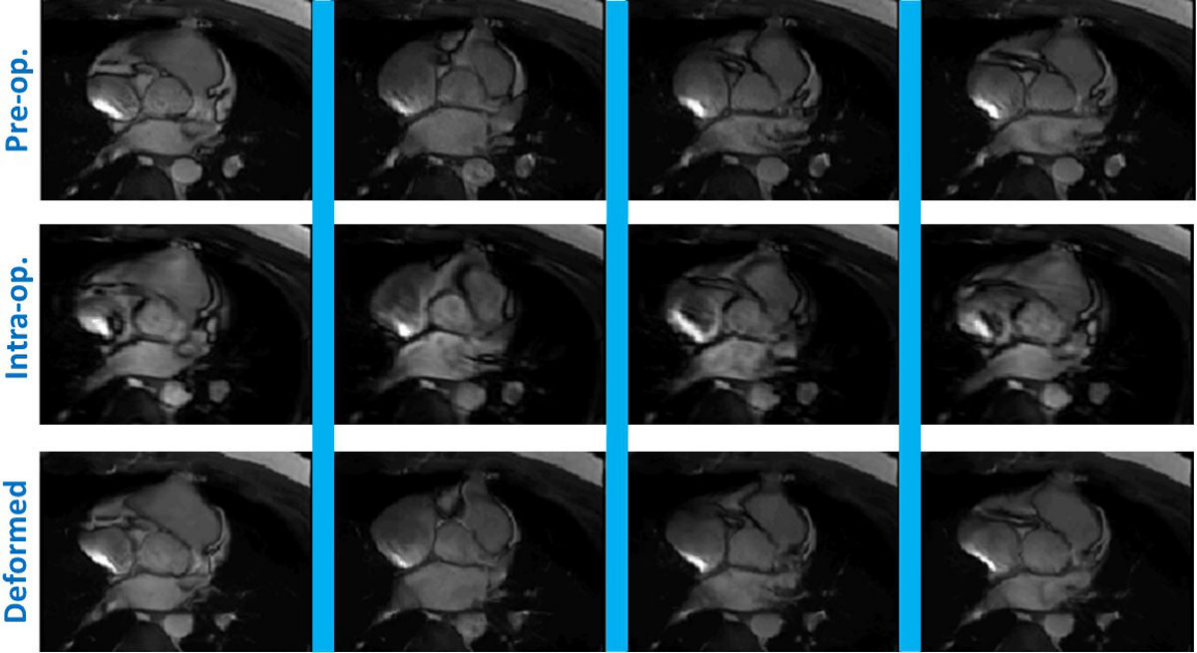
The deformed images co-registered between the intra-operative images (with scanning resolution: 66p*192s) and the pre-operative images (with 132p*192s). Both are on the same plane. Each Demons trial took less than 10 ms with the use of proposed computing architecture.

**Figure 2:**
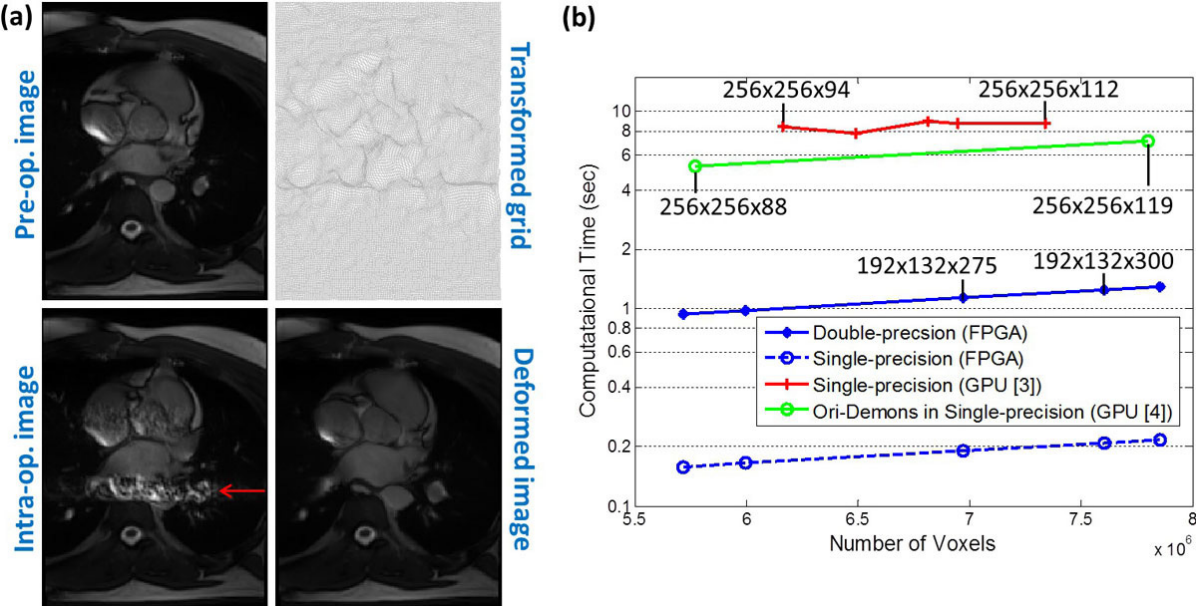
**(a) The intra-operative image interfered with motion artifacts (pointed by the red arrow)**. The deformed image was transformed by the grid applied to the pre-operative image; (b) Computational time of FPGA-based 3D Demons processed with single-and double-precision, compared with the graphics processing unit (GPU)-based Demons reported in [3] and [4]. Around 100 iterations were required to complete the Demons trials. Only [4] adopted original Demons force computation which involves fewer numbers of gradient operations.

Figure 1 shows Demons results of 2D cMRIs (Gradient Echo). Figure 2a shows a robust registration, even given the poor-quality intraoperative image with motion artifacts. The 3D Demons was applied to the corresponding images in 3D. An FPGA (Xilinx^® ^Virtex7-XC7V2000T) was used to investigate the accelerated performance. Figure 2b depicts the computational time required for the 3D images in various levels of resolution, > 40 times faster than the state-of-the-art acceleration techniques [3,4].

## Conclusions

The performance of the proposed computing architecture demonstrates its high potential for accelerating registration of 3D-gated MRI images to improve visualization of the MRI-guided cardiac therapy.

## Funding

NIH U41-RR019703, R43 HL110427-01, AHA 10SDG261039, EPSRC and Croucher Foundation Fellowship.
